# Prediction of survival in out-of-hospital cardiac arrest: the updated Swedish cardiac arrest risk score (SCARS) model

**DOI:** 10.1093/ehjdh/ztae016

**Published:** 2024-02-24

**Authors:** Pedram Sultanian, Peter Lundgren, Antros Louca, Erik Andersson, Therese Djärv, Fredrik Hessulf, Anna Henningsson, Andreas Martinsson, Per Nordberg, Adam Piasecki, Vibha Gupta, Zacharias Mandalenakis, Amar Taha, Bengt Redfors, Johan Herlitz, Araz Rawshani

**Affiliations:** Department of Molecular and Clinical Medicine, Institute of Medicine, University of Gothenburg, Wallenberg Laboratory, Blå stråket 5, staircase H, Sahlgrenska University Hospital, 413 45 Gothenburg, Sweden; Department of Molecular and Clinical Medicine, Institute of Medicine, University of Gothenburg, Wallenberg Laboratory, Blå stråket 5, staircase H, Sahlgrenska University Hospital, 413 45 Gothenburg, Sweden; Department of Cardiology, Sahlgrenska University Hospital, Blå stråket 5, Västra Götalands län, 413 45 Gothenburg, Sweden; Department of Molecular and Clinical Medicine, Institute of Medicine, University of Gothenburg, Wallenberg Laboratory, Blå stråket 5, staircase H, Sahlgrenska University Hospital, 413 45 Gothenburg, Sweden; Department of Molecular and Clinical Medicine, Institute of Medicine, University of Gothenburg, Wallenberg Laboratory, Blå stråket 5, staircase H, Sahlgrenska University Hospital, 413 45 Gothenburg, Sweden; Department of Clinical Medicine, Medicine Solna, Karolinska Institutet, Framstegsgatan, 171 64 Solna, Sweden; Department of Anesthesiology and Intensive Care, Sahlgrenska University Hospital, Blå stråket 5, 413 45 Gothenburg, Sweden; Department of Anaesthesiology and Intensive Care, Institute of Clinical Sciences, Sahlgrenska Academy, University of Gothenburg, Blå stråket 5, 413 45 Gothenburg, Sweden; Department of Anesthesiology and Intensive Care, Sahlgrenska University Hospital, Blå stråket 5, 413 45 Gothenburg, Sweden; Department of Anaesthesiology and Intensive Care, Institute of Clinical Sciences, Sahlgrenska Academy, University of Gothenburg, Blå stråket 5, 413 45 Gothenburg, Sweden; Department of Molecular and Clinical Medicine, Institute of Medicine, University of Gothenburg, Wallenberg Laboratory, Blå stråket 5, staircase H, Sahlgrenska University Hospital, 413 45 Gothenburg, Sweden; Department of Cardiology, Sahlgrenska University Hospital, Blå stråket 5, Västra Götalands län, 413 45 Gothenburg, Sweden; Center for Resuscitation Science, Department of Clinical Science and Education, Karolinska Institutets, Södersjukhuset, Jägargatan 20, staircase 1, 171 77 Stockholm, Sweden; Function Perioperative Medicine and Intensive Care, Karolinska University Hospital, Tomtebodavägen 18, 171 76 Stockholm, Sweden; Department of Anesthesiology and Intensive Care, Sahlgrenska University Hospital, Blå stråket 5, 413 45 Gothenburg, Sweden; Department of Anaesthesiology and Intensive Care, Institute of Clinical Sciences, Sahlgrenska Academy, University of Gothenburg, Blå stråket 5, 413 45 Gothenburg, Sweden; Department of Molecular and Clinical Medicine, Institute of Medicine, University of Gothenburg, Wallenberg Laboratory, Blå stråket 5, staircase H, Sahlgrenska University Hospital, 413 45 Gothenburg, Sweden; Department of Molecular and Clinical Medicine, Institute of Medicine, University of Gothenburg, Wallenberg Laboratory, Blå stråket 5, staircase H, Sahlgrenska University Hospital, 413 45 Gothenburg, Sweden; Department of Cardiology, Sahlgrenska University Hospital, Blå stråket 5, Västra Götalands län, 413 45 Gothenburg, Sweden; Department of Molecular and Clinical Medicine, Institute of Medicine, University of Gothenburg, Wallenberg Laboratory, Blå stråket 5, staircase H, Sahlgrenska University Hospital, 413 45 Gothenburg, Sweden; Department of Cardiology, Sahlgrenska University Hospital, Blå stråket 5, Västra Götalands län, 413 45 Gothenburg, Sweden; Department of Molecular and Clinical Medicine, Institute of Medicine, University of Gothenburg, Wallenberg Laboratory, Blå stråket 5, staircase H, Sahlgrenska University Hospital, 413 45 Gothenburg, Sweden; Department of Molecular and Clinical Medicine, Institute of Medicine, University of Gothenburg, Wallenberg Laboratory, Blå stråket 5, staircase H, Sahlgrenska University Hospital, 413 45 Gothenburg, Sweden; The Swedish Registry for Cardiopulmonary Resuscitation, Medicinaregatan 18G, 413 90 Gothenburg, Sweden; Department of Molecular and Clinical Medicine, Institute of Medicine, University of Gothenburg, Wallenberg Laboratory, Blå stråket 5, staircase H, Sahlgrenska University Hospital, 413 45 Gothenburg, Sweden; Department of Cardiology, Sahlgrenska University Hospital, Blå stråket 5, Västra Götalands län, 413 45 Gothenburg, Sweden; The Swedish Registry for Cardiopulmonary Resuscitation, Medicinaregatan 18G, 413 90 Gothenburg, Sweden

**Keywords:** Out-of-hospital cardiac arrest, Machine learning, Extreme gradient boosting, LightGBM

## Abstract

**Aims:**

Out-of-hospital cardiac arrest (OHCA) is a major health concern worldwide. Although one-third of all patients achieve a return of spontaneous circulation and may undergo a difficult period in the intensive care unit, only 1 in 10 survive. This study aims to improve our previously developed machine learning model for early prognostication of survival in OHCA.

**Methods and results:**

We studied all cases registered in the Swedish Cardiopulmonary Resuscitation Registry during 2010 and 2020 (*n* = 55 615). We compared the predictive performance of extreme gradient boosting (XGB), light gradient boosting machine (LightGBM), logistic regression, CatBoost, random forest, and TabNet. For each framework, we developed models that optimized (i) a weighted F1 score to penalize models that yielded more false negatives and (ii) a precision–recall area under the curve (PR AUC). LightGBM assigned higher importance values to a larger set of variables, while XGB made predictions using fewer predictors. The area under the curve receiver operating characteristic (AUC ROC) scores for LightGBM were 0.958 (optimized for weighted F1) and 0.961 (optimized for a PR AUC), while for XGB, the scores were 0.958 and 0.960, respectively. The calibration plots showed a subtle underestimation of survival for LightGBM, contrasting with a mild overestimation for XGB models. In the crucial range of 0–10% likelihood of survival, the XGB model, optimized with the PR AUC, emerged as a clinically safe model.

**Conclusion:**

We improved our previous prediction model by creating a parsimonious model with an AUC ROC at 0.96, with excellent calibration and no apparent risk of underestimating survival in the critical probability range (0–10%). The model is available at www.gocares.se.

## Graphical Abstract

**Graphical Abstract:**
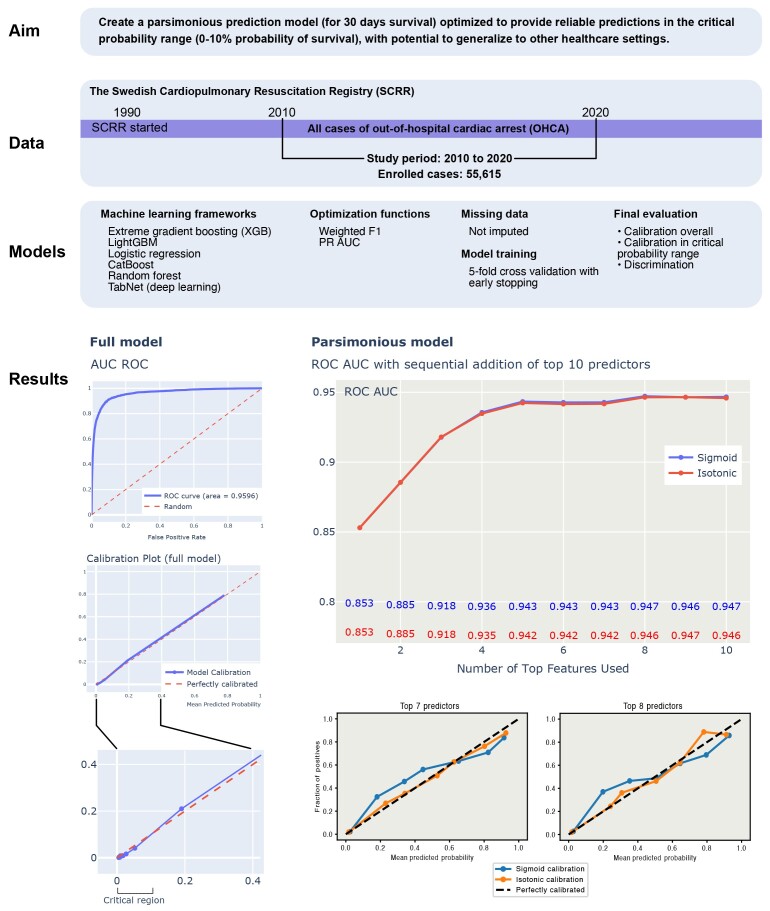

## Introduction

Out-of-hospital cardiac arrest (OHCA) is a substantial public health concern worldwide.^1,2^ Despite improvements in resuscitation and increased rates of bystander cardiopulmonary resuscitation (CPR), survival following an OHCA is still approximately only 10%,^2^ with the majority dying prior to hospital arrival.^3,4^ However, the rate of return of spontaneous circulation (ROSC) is roughly three-fold the survival rate.^2^ This is explained by the fact that the myocardium can endure longer periods of hypoxia and anoxia, when compared with neurons.^5^ Ultimately, the majority of patients admitted to hospital after an OHCA will succumb due to irreversible ischaemic and reperfusion-induced brain damage.^6^

Several critical decisions must be made rapidly during OHCA. The first decision is whether to initiate and continue CPR.^7^ There are guideline criteria for termination of resuscitation (TOR), and these include information on witnessed status, bystander CPR, pre-hospital ROSC, and defibrillations.^8^ These criteria fail to prognosticate the vast majority of patients, namely those who are not immediately resuscitated or fulfilled TOR criteria. Predicting survival is difficult during OHCA.^9^ The human error and tendency for biased judgements is an obvious culprit. Statistical models are key to improving survival predictions. These models must be parsimonious, have excellent discrimination and calibration, as well as generalize well to other populations.

We recently published a prediction model for OHCA outcomes.^10^ The model showed overall excellent discrimination and likewise good overall calibration. However, there was room for improvement. Hyperparameter tuning was less comprehensive than desired; the optimization function was not cost-sensitive, and we imputed missing values (which will impair the ability to generalize and allow for real-world scenarios). This study addresses these shortcomings and produces a prediction model that is readily accessible as an open Application Programming Interface (API), ensuring that anyone can utilize and validate the model.

## Methods

### Study population

We used the Swedish Cardiopulmonary Resuscitation Registry (SCRR), which records all cases of OHCA in Sweden.^11–13^ The SCRR is a national quality registry that has monitored OHCA in Sweden since 1990. All cases, matching inclusion, are registered and aligned with the Utstein style of reporting for variables and outcomes, enabling an immensely detailed description for each patient. This study included cases enrolled from 2010 to 2020 (*n* = 55 615). The SCRR encompasses virtually all OHCA patients in whom resuscitation was attempted by Emergency Medical Services.

### Data merger

The SCRR was merged with the Swedish National Patient Registry. This enabled the retrieval of diagnoses using International Classification of Diseases 10th revision (ICD-10) codes, from both inpatient and outpatient clinics back to the year 2001. The ICD chapters available in the merged data set included Chapters A through Z. We also merged the SCRR with the Prescribed Drug Registry, where data were collected from 2005, in order to obtain information about prescription for drugs in Anatomical Therapeutic Chemical Sections A (alimentary tract and metabolism), B (blood and blood-forming organs), and C (cardiovascular system). Information regarding vital status was obtained from the cause of death registry.

### Data pre-processing

During data pre-processing, we removed variables with zero variance (ICD codes C97, G98, V03, M17, Z13, T03, D05, and L03). Additionally, we removed specific hospital units (as it will not be generalizable), geographical district, the presumed cause of cardiac arrest (since that information is uncertain at the point of the intended use of the prediction model), and details regarding the location of cardiac arrest (e.g. type of public place, etc.). Moreover, variables depicting ICD Sections S, T, W, V, X, Y, U, and Z were omitted as they were deemed irrelevant, as per our previous experience modelling the data.

### Machine learning frameworks

We explored extreme gradient boosting (XGB), LightGBM (Microsoft’s implementation of gradient boosting), logistic regression, CatBoost, random forest, and Google’s TabNet. The latter was the only neural network tested. Logistic regression, random forest, and CatBoost were consistently inferior to XGB and LightGBM in several initial tests with hyperparameter tuning. TabNet showed excellent calibration but inferior discrimination when compared with XGB and LightGBM, and was ultimately excluded since it lacks intrinsic modelling of missing data. Hence, comprehensive model development was done for XGB and LightGBM. Extreme gradient boosting and LightGBM differ slightly with regard to their handling of categorical predictors, tree growth, handling of missing variables, and regularization. Details on XGB^14^ and LightGBM can be found elsewhere.^15^

### Data splitting

The data set was divided into two subsets: the initial split designated 70% of the data for preliminary training and validation, and the remaining 30% was reserved for final testing. Of the former 70%, 80% was used for training and 20% for validation.

### Outcome

The outcome was 30-day survival after OHCA.

### Initial feature selection

A total of 384 features (candidate predictors) were available. Reducing the number of predictors is necessary to reduce noise, derive a parsimonious model, increase generalizability, reduce overfitting, and alleviate the computational burden. For initial feature screening, a basic LightGBM model was deployed to identify the most influential predictors of the outcomes. The basic model used 1500 trees, and the learning rate was fixed at 0.025 with a maximum tree depth of 10. Adjusting other hyperparameters did not materially affect the rank order of feature importance. Upon fitting this model on the training data, the top 40 predictors were extracted. Similarly, a basic XGB model was utilized for an initial feature selection. The model was set up with 1500 trees and a maximum depth of 10, and the learning rate was set at 0.01. A similar extraction of the top 40 predictors, based on their feature importance, was performed. While this initial feature selection did not employ hyperparameter tuning, multiple repeated runs with different hyperparameters showed no difference in the top predictors.

During development, we computed >5000 LightGBM and XGB classifiers each, with hyperparameter tuning performed using the Optuna framework.^16^ Using the optimization history, we gradually tapered the hyperparameter grid and used fewer trials (for computational reasons). Hence, we present results here for one of the runs with 1000 models for each strategy.

We used two modelling strategies for XGB and LightGBM. The first strategy used a weighted F1 score to optimize the hyperparameters. The weighted F1 was selected to penalize models that yielded more false negatives (higher false-negative rate), as false-negative predictions of survival may be fatal with the intended use of the model. The second strategy used a precision–recall area under the curve (PR AUC) as the optimization parameter. All models used five-fold cross-validation and early stopping.

The purpose of the weighted F1 score was to place a higher penalty for misclassifying survivors, to prioritize their correct classification. In the context of imbalanced data sets, where the positive class (survival) is the minority class, a PR AUC is an informative metric compared with the traditional AUC ROC. The AUC ROC may provide an overly optimistic view of model performance as it takes into account the true negative rate, which is likely to be high when the negative class is the majority. Precision–recall area under the curve, on the other hand, focuses on the performance of the classifier on the positive (minority) class, making it a more suitable metric for our purpose.

For each strategy, the best model was used to retrain the model on the full training set and then used to test the model. We then assessed the AUC ROC and calibration curves for the full model (all 40 predictors) as well as the relative importance of the included features. This will indicate the opportunity of using a parsimonious model. We considered a model with 10 predictors as the largest reasonable model for a globally useful prediction model. Therefore, we retrained the best model using top 1 to top 10 predictors, sequentially added, and assessed the AUC ROC after isotonic and sigmoid calibration. The purpose was to evaluate how many predictors are necessary to attain a high performance. We finally evaluated the calibration of each of these models (using 1 to a maximum of 10 predictors).

### Ethics

This study follows the principles of the Declaration of Helsinki and has received approval from the Swedish Ethical Committee (No. 2020-02017).

## Results

Patient characteristics are presented in *Table 1*. A total of 55 615 patient cases were studied. The mean age was 69 years, and 66% were males. An initial shockable rhythm was noted in 23.2%, and in 33.3% of patients, defibrillation was attempted, while 78.8% received epinephrine. Spontaneous circulation on arrival at the emergency department (ED) was noted in 94.5% of those who survived to 30 days, compared with 33.5% among those who did not survive to 30 days.

**Table 1:** Baseline characteristics of 55 615 individuals experiencing an out of-hospital cardiac arrest in Sweden during 2010–20

		All	Dead	Survived
Subjects, *n*		55 615	49 424	6191
Age, mean (SD)		68.9 (17.9)	69.8 (17.6)	61.2 (18.7)
Sex, *n* (%)	Male	36 645 (66.0)	32 012 (64.9)	4633 (75.0)
Cardiac arrest in public place, *n* (%)	Home	39 661 (71.6)	36 817 (74.8)	2844 (46.2)
	Public place	8989 (16.2)	6952 (14.1)	2037 (33.1)
	Other place	6728 (12.1)	5453 (11.1)	1275 (20.7)
Cardiac arrest during sports activity, *n* (%)		616 (2.8)	389 (2.0)	227 (9.0)
Time from cardiac arrest to CPR start, median (Q1, Q3)		3.0 (0.0, 10.0)	3.0 (0.0, 10.0)	1.0 (0.0, 3.0)
Time from cardiac arrest to ambulance arrival, median (Q1, Q3)		13.0 (8.0, 20.0)	13.0 (8.0, 20.0)	9.0 (6.0, 15.0)
Time from cardiac arrest to first defibrillation, median (Q1, Q3)		15.0 (8.0, 24.0)	17.0 (11.0, 26.0)	8.0 (2.0, 13.0)
Initial rhythm—ventricular fibrillation/pulseless ventricular tachycardia, *n* (%)	Asystole	29 248 (59.6)	28 745 (64.8)	503 (10.8)
	PEA*	8421 (17.2)	7989 (18.0)	432 (9.2)
	VF**/pVT***	11 374 (23.2)	7633 (17.2)	3741 (80.0)
Bystander CPR education, *n* (%)	Layperson, educated in CPR	4215 (36.7)	3711 (36.7)	504 (36.7)
	Layperson, not educated in CPR	5389 (46.9)	4882 (48.2)	507 (36.9)
	Healthcare personnel	1891 (16.5)	1529 (15.1)	362 (26.4)
Defibrillation performed, *n* (%)		17 812 (33.3)	13 799 (29.1)	4013 (66.4)
Cordarone administered, *n* (%)		6365 (11.8)	5433 (11.3)	932 (15.6)
Epinephrine administered, *n* (%)		43 290 (78.8)	41 130 (84.1)	2160 (35.8)
Spontaneous circulation on arrival, *n* (%)		14 190 (44.7)	8705 (33.5)	5485 (94.8)
Previous acute myocardial infarction, *n* (%)		7537 (13.6)	6794 (13.7)	743 (12.0)
Previous heart failure, *n* (%)		12 669 (22.8)	11 716 (23.7)	953 (15.4)
Previous Type 2 diabetes, *n* (%)		10 609 (19.1)	9887 (20.0)	722 (11.7)
Previous aortic stenosis, *n* (%)		3377 (6.1)	3147 (6.4)	230 (3.7)

The columns ‘dead’ and ‘survived’ refer to survival status at 30 days post-OHCA.

CPR, cardiopulmonary resuscitation; *PEA, pulselsess electrical activity; **VF, ventricular fibrillation; ***pVT, pulselsess ventricular tachycardia.

**Figure 1:**
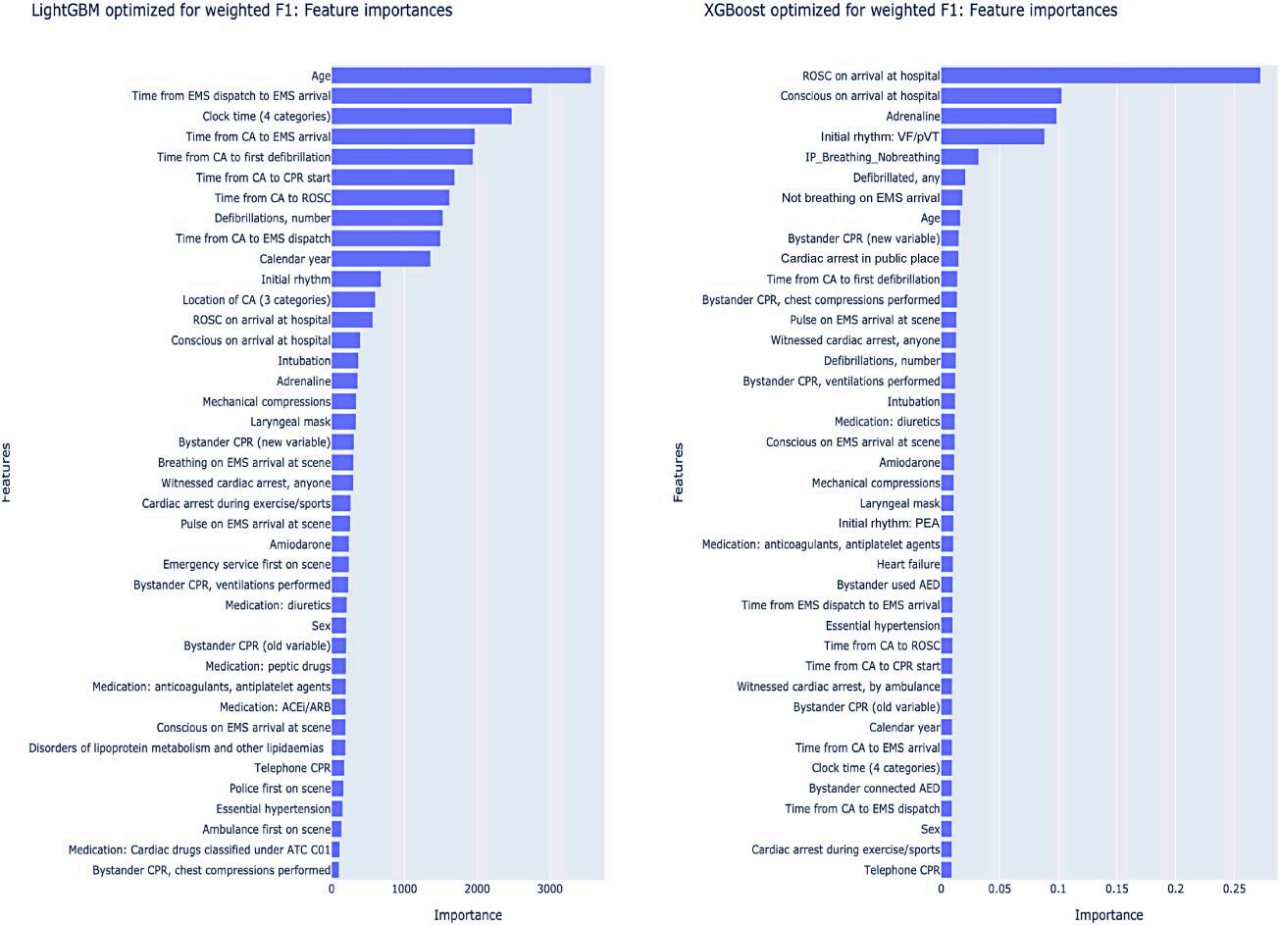
Variable importance in initial feature screening. Differences in feature importance were observed between LightGBM (left) and extreme gradient boosting (right), in terms of both the order of the features and the distribution of importances. For LightGBM, a larger number of variables exhibited higher importance values. In contrast, for extreme gradient boosting, the top five predictors dominated the importance, with the single strongest predictor being ROSC on arrival in the emergency department.

*Figure 1* shows the difference in feature importance between LightGBM and XGB. The order and distribution of feature importance differed for each model. LightGBM assigned higher importance values to a broader set of variables. In contrast, XGB’s feature importance was concentrated, with the top five predictors being predominantly influential. Notably, the most significant predictor in XGB was ROSC upon arrival in the ED.

**Figure 2:**
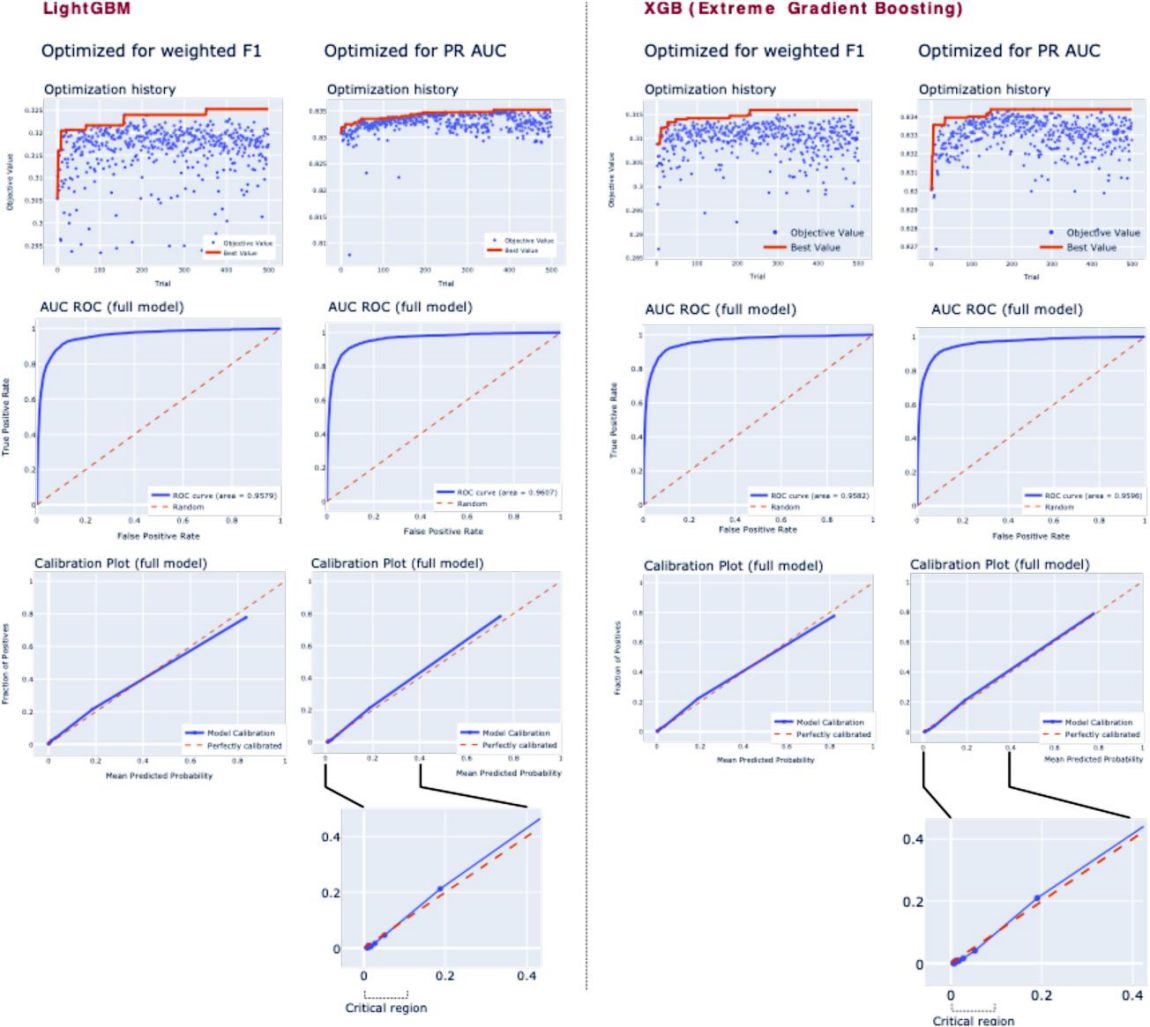
An optimization history of an AUC ROC and calibration plots for all strategies. This figure presents results from the optimization history, focusing (first row) the AUC ROC for the full model (encompassing all 40 predictors) alongside the calibration for the full model. The AUC ROC scores for LightGBM were 0.958 (weighted F1) and 0.961 (a precision–recall area under the curve) and for extreme gradient boosting, 0.958 (weighted F1) and 0.960 (the precision–recall area under the curve), respectively. Thus, the full models were very similar in terms of discriminatory power. The calibration plots show a very small tendency to underestimate survival for the LightGBM models and the opposite for the extreme gradient boosting models. The best model in the critical range (0–10% likelihood of survival) was noted for extreme gradient boosting optimized with the precision–recall area under the curve.

*Figure 2* shows optimization histories, AUC ROC, and calibration plots across all strategies (full model encompassing 40 predictors). The AUC ROC scores for LightGBM were 0.958 (optimized using weighted F1) and 0.961 (optimized using a PR AUC), while for XGB, the respective scores were 0.958 and 0.960, demonstrating very close similarity in discriminatory power between the models. The calibration plots (*Figure 2*, third row) indicate a subtle underestimation of survival in the LightGBM models, contrasting with a mild overestimation for XGB. In the crucial range of 0–10% likelihood of survival, the XGB model, optimized with the PR AUC, emerges as the most clinically safe model, given a very small tendency to overestimate survival in that range.

**Figure 3:**
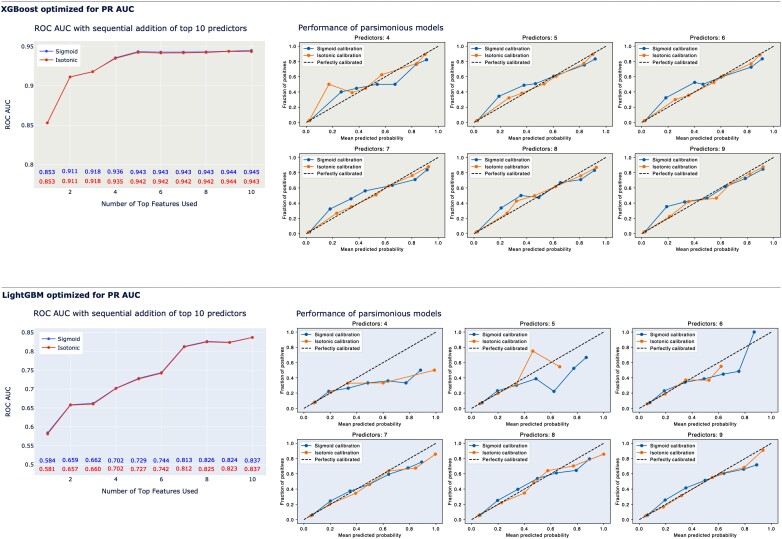
AUC ROC and calibration plots for LightGBM and extreme gradient boosting optimized using a precision–recall area under the curve.

*Figure 3* shows how the AUC ROC (left, large panels) and calibration (right, small panels) are affected by sequentially adding the top predictors. These results pertain to models optimized using the PR AUC. As evident, a marked ascent in the AUC ROC was observed for XGB with the inclusion of a few predictors, as anticipated based on feature importance, allowing XGB with five predictors to markedly surpass LightGBM in terms of discriminatory capacity. However, the parsimonious XGB models underestimated survival in the critical range (0–10%) when using fewer than eight predictors. LightGBM exhibited better calibration.

**Figure 4:**
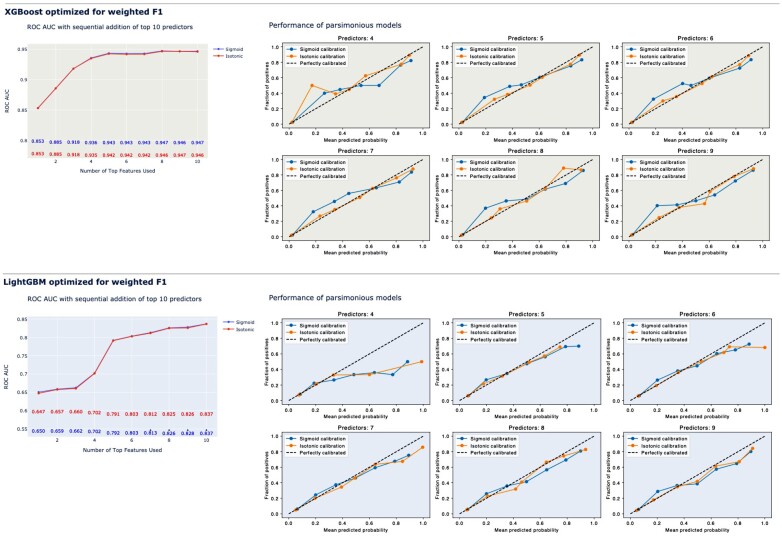
AUC ROC and calibration plots for LightGBM and extreme gradient boosting optimized using weighted F1.

*Figure 4* shows corresponding plots for models optimized using weighted F1. With five predictors, the AUC ROC was 0.94 for XGB compared with 0.79 for LightGBM. However, XGB models with fewer than eight predictors underestimated survival. The underestimation was subtle for seven predictors and negligible with eight or nine predictors. For LightGBM, the discrimination (AUC ROC) never surpassed 0.85 when testing the top 1 to top 10 predictors, although calibration was slightly better than for XGB.

## Discussion

Our group recently published a prediction model for OHCA outcomes.^10^ The model exhibited excellent discrimination but lacked an assessment of the calibration for parsimonious models, as well as other shortcomings mentioned above. We now improved our previous approach, providing more comprehensive hyperparameter tuning and cost-sensitive model development, allowed for missing data to better reflect reality, and carefully assessed the calibration performance with the parsimonious models. We present a final model (XGB with eight predictors optimized using weighted F1) that has the necessary characteristics for a model intended for early prognostication of survival after OHCA. The model is readily accessible as an open API, ensuring that anyone can utilize and validate the model in other settings.

Decision-making in life-or-death scenarios is a difficult challenge, even for highly experienced clinicians. In this context, mathematical prediction models offer important tools to support and enhance the clinical decision-making processes. While existing guidelines do provide criteria for the TOR, a substantial portion of all patients fall outside the TOR criteria. The rapid integration of mathematical prediction models into hospital and pre-hospital systems will inevitably make prediction models like SCARS common, and therefore, it is imperative for clinicians to understand the nuances and limitations lurking beneath AUC ROC values. We emphasize that prediction models for OHCA prognostication must maintain robust discriminatory capacity and exceptional calibration within a specific likelihood range (0–10% likelihood of survival). The latter is critical as this interval is where decisions are difficult. Furthermore, we considered the implications of false-negative misclassification in this setting. Given the complexity of these concepts and the rapid introduction of machine learning in clinical practice, clinicians would benefit greatly from familiarizing themselves with these parameters and how they relate to their clinical decisions.

With regard to the current model, referred to as SCARS-2, we believe it reliably calculates the probability of survival at 30 days. Given that it was trained and evaluated in a nationwide registry, it does not require any further validation for the Swedish environment, although it should undergo prospective retraining and calibration in order to incorporate temporal changes in population characteristics and cardiac arrest care. For other countries, however, we recommend that researchers utilize our open API (www.gocares.se) to evaluate the prediction model on local data. Researchers and clinicians should be aware that deployment of the model as a clinical tool requires regulatory approval in Europe.

LightGBM and XGB both displayed an excellent AUC ROC (whether optimized using weighted F1 or the PR AUC). However, XGB was found to be the overall best model because it offered the highest discriminatory capacity at a similar calibration. The final model utilized eight predictors, with an AUC ROC of 0.94 and reliable calibration in the critical interval (0–10% likelihood of survival). We believe that these prediction models should perform particularly well in the likelihood interval where they may affect management, which is in the lower likelihood range.

We believe that the ED setting, the employment of advanced prediction models, such as the one developed in this study, holds particular promise in guiding clinical decision-making, including the withdrawal of interventions. The high-pressure environment in the ED often sees prolonged interventions, occasionally resulting in the ROSC despite biological death having been manifested. This scenario leaves clinicians in a quandary, often hesitant to terminate resuscitation efforts despite bleak prospects, a decision further compounded by their limited exposure to a high volume of cardiac arrest cases necessary for developing the judgement and skills. By providing a more accurate and reliable estimation of survival likelihood, the model aids in avoiding the unnecessary prolongation of interventions, enhancing resource allocation, and ensuring the delivery of appropriate and ethically sound care. Nevertheless, these models must be viewed as a compliment to clinical judgement and reasoning and not as a replacement. Indeed, these models can mitigate the impact of human biases, enhance the accuracy of survival predictions post-OHCA, and guide more informed, evidence-based clinical decisions.

Other researchers have contributed significantly to this topic. Seki *et al*.^17^ used random forest for predicting outcomes of OHCA with presumed cardiac aetiology. The study used data from the SOS-KANTO 2012 study, which included 16 452 patients. The model showed high accuracy in predicting 1-year survival but was limited to one aetiology, and data are arguably outdated by now. Kwon *et al*.^18^ developed and validated a deep learning model for OHCA prognostication. They predicted neurological recovery and survival to discharge for patients who experienced ROSC after OHCA. Similarly, Pareek *et al*.^19^ and Adrie *et al.*^20^ also created prediction models for neurological outcomes after OHCA. Maupain *et al*.^21^ introduced the Cardiac Arrest Hospital Prognosis score, a tool developed to stratify patients admitted to the intensive care unit after experiencing an OHCA, also with an emphasis on neurological outcomes.^22^

### Limitations

Our model requires regulatory approval before implementation in routine clinical practice. Our model is intended only to augment or assist the clinician’s judgement. Since our model included virtually all cases of OHCA in Sweden, it can be viewed as reliable within the Swedish healthcare setting. However, external validation in other healthcare settings must be performed to evaluate the generalizability of the model. Such validation should imply using retrospective data. Ideally, a randomized trial with the intention of confirming the safety of the prediction model should be performed. Until such trials have been conducted, we argue that, given the increasing prevalence of prediction models for cardiac arrest care, international organizations such as the International Liaison Committee on Resuscitation (ILCOR), the European Resuscitation Council (ERC), and the American Heart Association (AHA) should review the existing models and their underlying evidence to put forward practice recommendations. We advise against using this prediction model to evaluate cases due to drowning, cases with severe hypothermia, or pregnancy cases, since the prediction model did not include those variables during training. Finally, there are special circumstances (e.g. thrombo-embolic cardiac arrests, sepsis, etc.) for which there are guideline recommendations with regard to the resuscitation protocol, which should be applied regardless of predictions from a mathematical model.

## Conclusions

We improved our previous OHCA prediction model, using a large and nationwide training data set, to achieve a model with an AUC ROC at 0.96, which exhibited excellent calibration and no apparent risk of underestimating survival in the critical probability range (0–10%). The model relies on a few predictors that are all available worldwide, and it is freely available via an online API at www.gocares.se.

## Data Availability

Data is available after appropriate ethical approvals have been obtained.
